# Occupational therapy intervention in a mental health hospitalization unit

**DOI:** 10.1192/j.eurpsy.2025.1633

**Published:** 2025-08-26

**Authors:** M. Valverde Barea, M. N. Romero Muñoz, L. Cerón Lorente

**Affiliations:** 1 Psychiatry, H. U Jaén, Jaén; 2 Psychiatry, H. Jerez de la Frontera, Jerez de la Frontera, Spain

## Abstract

**Introduction:**

Patients with severe mental disorders (SMD) often have a high number of admissions and readmissions, which leads to a loss of quality of life and functionality. The approach to patients must be multidisciplinary with the intervention of psychiatry, psychology and occupational therapy. Occupational Therapy is an important tool in acute mental health units; in the literature it has demonstrated its effectiveness in preventing suicide, offering a person-centered approach, contributing to improving their well-being and quality of life. Also noteworthy are the occupational therapy therapeutic groups for health education; sleep-wake balance; cognitive rehabilitation, social skills and relaxation that can be carried out in hospitalization units and demonstrate significant improvement in hospitalized SMD patients.

**Objectives:**

To describe the importance of the joint approach with occupational therapy in the acute mental health unit by describing 2 clinical cases.

**Methods:**

A 54-year-old male was admitted to the mental health hospitalization unit involuntarily due to manic decompensation. During the examination, he was in a euphoric mood and reported a decrease in the need for sleep. He spent all of his pension on compulsive shopping. The family said that in the weeks prior to admission, the patient had made abrupt decisions and had a violent attitude. He has now abandoned treatment. A 56-year-old woman, diagnosed with schizoaffective disorder, was admitted due to psychotic decompensation after being isolated at home for 1 month, with neglect of hygiene and inadequate nutrition due to thoughts of poisoning and neglect of pharmacological treatment. Upon examination, she said he was delusional about harm and poisoning, isolation, neglect of self-care.

**Results:**

After the intervention by the therapeutic team, psychopharmacological treatment was reintroduced and daily attendance at occupational therapy groups was indicated, after which the patient showed a notable improvement. We conclude that occupational therapy has allowed a faster recovery, together with the proper intake of medication, constituting an effective therapeutic approach in the treatment. Recovering the deficient Basic Activities of Daily Living related to hygiene and personal image, adherence to treatment and social relationships. Upon discharge, both patients participate in the rehabilitation unit and continue specific programs.

**Conclusions:**

The intervention with psychoeducational programs by Occupational Therapy and the multidisciplinary team in the acute unit improves disease awareness, health knowledge and reduces the rate of readmission in patients with SMD.

**Disclosure of Interest:**

None Declared

